# Validation of De Novo Peptide Sequences with Bottom-Up Tag Convolution

**DOI:** 10.3390/proteomes10010001

**Published:** 2021-12-29

**Authors:** Kira Vyatkina

**Affiliations:** 1 Laboratory of Bioinformatics and Mathematical Biology, Alferov University, 194021 St. Petersburg, Russia; vyatkina@spbau.ru; 2 Laboratory of Neuroscience and Molecular Pharmacology, Institute of Translational Biomedicine, Saint Petersburg State University, 199034 St. Petersburg, Russia; 3 Department of Software Engineering and Computer Applications, Faculty of Computer Science and Technology, Saint Petersburg Electrotechnical University “LETI”, 197022 St. Petersburg, Russia

**Keywords:** tandem mass spectrometry, de novo sequencing, tag convolution

## Abstract

De novo sequencing is indispensable for the analysis of proteins from organisms with unknown genomes, novel splice variants, and antibodies. However, despite a variety of methods developed to this end, distinguishing between the correct interpretation of a mass spectrum and a number of incorrect alternatives often remains a challenge. Tag convolution is computed for a set of peptide sequence tags of a fixed length k generated from the input tandem mass spectra and can be viewed as a generalization of the well-known spectral convolution. We demonstrate its utility for validating de novo peptide sequences by using a set of those generated by the algorithm PepNovo+ from high-resolution bottom-up data sets for carbonic anhydrase 2 and the Fab region of alemtuzumab and indicate its further potential applications.

## 1. Introduction

Tandem mass spectrometry (MS/MS) has established itself as the dominant technique in proteomics. First recognized as such was the more elaborated bottom-up technology, which analyzes peptides resulting from protein enzymatic digestion; however, the recently emerged top-down approach that analyzes intact proteins is nowadays rapidly gaining popularity.

Analysis of MS/MS spectra acquired from peptides or proteins often amounts to a consideration of pairwise differences of peak masses rather than those masses on their own. For instance, pairs of peaks separated by the amino acid masses give rise to edges in a spectrum graph [1,2], and ladders of such peaks define peptide sequence tags [3], which have become the basis of several methods for peptide and protein identification from database search [3,4,5,6,7,8,9,10,11,12,13,14,15,16,17,18] and also proved to be useful for limiting the number of de novo sequence possibilities [19,20]. The key step that precedes deisotoping and charge state deconvolution of MS/MS spectra is the detection of (candidate) isotopomer envelopes, the theoretical counterparts of which are represented by groups of equally spaced peaks [21,22,23,24,25,26,27]. A more sophisticated example is given by spectral convolution [28], which examines pairwise differences between the masses of peaks picked up from two distinct spectra, along with their multiplicities (i.e., the number of times they are observed) in order to estimate similarity between the latter. The key observation behind is that the multiplicity of zero equals the number of peaks the two spectra have in common, or *shared peaks count* for those, and presence of a few non-zero values with high multiplicities likely indicates that the spectra were acquired from two peptides that are a few mutations apart.

In [29], we introduced the notion of *tag convolution* for a top-down LC-MS/MS dataset, which may be viewed as generalization of spectral convolution, and it is computed across the entire set of input spectra—or, more precisely, over a set of sequence tags generated from those spectra. We demonstrated that tag convolution can be efficiently used for combining together protein sequence fragments generated by the Twister de novo sequencing algorithm from top-down MS/MS data; as a result, we obtained so-called *gapped strings*, in which the missing portions of the sequence were substituted by their masses. We also mentioned that the concept of tag convolution could be adapted to the bottom-up case and applied for validating de novo peptide sequences. However, to this end, it is essential to take into account that the number of tags derived from a typical bottom-up data set will be a few orders of magnitude larger than in the top-down case and that the pairs of tags matching the same peptide will have relatively close mass offsets.

Recall that for a spectrum *S*, a peptide sequence tag of length *k*, or *k*-tag, is defined by k+1 peaks of *S* separated by the amino acid masses; the respective amino acids spell out the *tag string*, and the mass of the leftmost peak determines the mass offset, or simply *offset*, assigned to the resulting tag. Given a set T of *k*-tags extracted from a set of input MS/MS spectra and two *k*-mers $w_{1}$ and $w_{2}$, tag convolution computes offset differences for the pairs of tags from T labeled with $w_{2}$ and $w_{1}$, respectively, and along with each encountered value, it reports its multiplicity equal to the number of pairs of tags that contributed to it. The intuition behind is that if $w_{1}$ precedes $w_{2}$ in the sequence *s* of a protein or peptide subject to analysis, then the mass of the subsequence of *s* starting at the beginning of $w_{1}$ and ending right before $w_{2}$ will, thus, become registered with high multiplicity.

For this approach to work as expected, it is crucial that most of the tags composing T be correct. In order to ensure this holds, we employ the tag generation strategy introduced in [30] for the case of top-down MS/MS spectra and apply it to bottom-up MS/MS spectra collected at a high resolution [31]. It first deconvolutes the input spectra with MS-Deconv [27] and subsequently generates *k*-tags applying ultra-low constant mass tolerance of 4 mDa, thus profiting from the fact that while an absolute error in a peak mass (especially a large one) can be accordingly large, the difference in those corresponding to consecutive fragment ions tends to be substantially smaller.

In what follows, we provide a formal definition of bottom-up tag convolution, describe a procedure that uses it for validating de novo amino acid sequences, and illustrate its performance on bottom-up datasets for carbonic anhydrase 2 (CAH2) and alemtuzumab. We conclude by indicating future methods for developing this concept.

## 2. Materials and Methods

### 2.1. Generation of k-Tags

The input MS/MS spectra acquired at a high resolution are first deconvoluted, to which end we use MS-Deconv [27]. Let S denote the resulting set of deconvoluted spectra.

Subsequently, we extract from each spectrum S∈S a number of high-quality *k*-tags for a fixed length *k*. This is accomplished by means of the method first proposed in [30] for top-down MS/MS spectra and later successfully applied to bottom-up data [31]. First, a spectrum graph $G_{S}$ is constructed for *S*. Its vertices correspond to the peaks from *S* and are scored with underlying peak intensities; a directed edge $\bar{uv}$ is introduced between two vertices *u* and *v* if Mass(v)>Mass(u), and Mass(v)−Mass(u) equals the mass of some amino acid *a* up to a predefined tolerance, where Mass(u) and Mass(v) denote the masses of the peaks from *S* that gave rise to *u* and *v*, respectively. Thereby we rely upon the observation that peaks with nearby masses typically bear a similar error in those; therefore, the relative mass difference for two peaks corresponding to consecutive fragment ions should be highly accurate. Thus, for a small ε denoting the allowable deviation from the “anticipated” peak mass (which we expect to differ from the theoretical one by a certain value depending on the absolute mass), we check whether Mass(v)−Mass(u)<2ε, and, if so, create an edge $\bar{uv}$ and label it with *a*. Based on automated and manual analysis of a few datasets, we set ε to 4 mDa and kept this value throughout all our experiments.

Next, an optimal path (with respect to the vertex scores) is computed for each connected component of $G_{S}$, and all the possible *k*-tags are derived from it; note that two tags with a same amino acid string and offset originating from distinct spectra are considered different. In this manner, we obtain a set T=T(S) of *k*-tags, based on which tag convolution is further computed.

### 2.2. Bottom-Up Tag Convolution

When describing computation of bottom-up tag convolution, we will generally follow the scheme from [29]. However, the masses potentially separating pairs of tags under consideration will be analyzed and processed in a distinct manner as compared to the top-down case due to the following reasons:

- The number of tags originating from the same peptide is typically quite large;
- The mass offsets of tags matching the same peptide are usually close; thus, their differences are accurate;
- Unlike in the top-down case, ±1 Da deconvolution errors are rarely observed in the bottom-up MS/MS spectra.

For a tag t∈T, let s(t) and o(t) denote its amino acid string and offset, respectively. Moreover, let K=K(T) denote the set of tag strings induced by T:

K={w∣∃t∈T:s(t)=w}.

**Figure 1:**
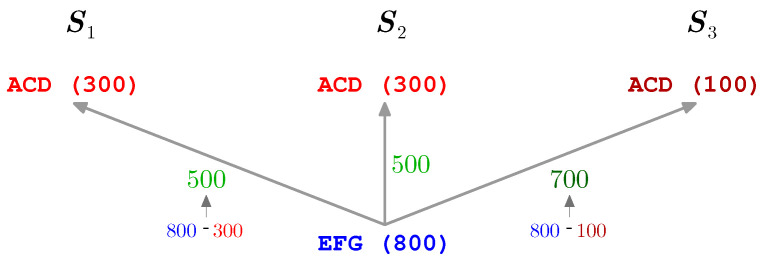
For a toy input set $S=\{S_{1},S_{2},S_{3}\}$, spectra $S_{1}$, $S_{2}$, and $S_{3}$ contain one, two, and one 3-tag(s), respectively. Here, τ(ACD,EFG)={(500,2),(700,1)}.

For two *k*-mers $w_{1},w_{2}\in K$, tag convolution $\tau (w_{1},w_{2})$ examines each pair $\left(t_{1},t_{2}\right)$ of tags from T labeled with $w_{1}$ and $w_{2}$, respectively, and computes difference $o\left(t_{2}\right)-o\left(t_{1}\right)$ of their associated offsets. Its output represents the set of observed values $d_{i}$, each endowed with the multiplicity $m_{i}$ being equal to the number of pairs of tags that produced it (up to a predefined tolerance): $\tau \left(w_{1},w_{2}\right)=\left\{\left(d_{i},m_{i}\right)|1\leq i\leq h\right\}$, where *h* is the number of offset differences encountered. If either $w_{1}$, or $w_{2}$, or both do not belong to K, the output of $\tau (w_{1},w_{2})$ is an empty set. A toy example illustrating this concept is provided in Figure 1.

Observe that spectral convolution [28] of two spectra $S_{1}$ and $S_{2}$ constitutes a special case of tag convolution for $S=\{S_{1},S_{2}\}$ and all the possible 0-tags—i.e., peaks from $S_{1}$ and $S_{2}$—upon a convention that each 0-tag derived from $S_{1}$ and $S_{2}$, respectively, has been assigned an artificial label $z_{1}^{*}$ and $z_{2}^{*}$, respectively, where $z_{1}^{*}$ and $z_{2}^{*}$ are distinct.

Intuitively, it should be expected that if the *k*-mers $w_{1}=a_{i}\ldots a_{i+k-1}$ and $w_{2}=a_{j}\ldots a_{j+k-1}$ represent two substrings of the sequence $s=a_{1}\ldots a_{n}$ of a target peptide *P*, where 1≤i<j≤n−k+1, and are unique with respect to the sequences of all the peptides subject to analysis (up to reversal), then the offset difference approximately equal to $Mass(a_{i}\ldots a_{j-1})$ will appear in the output of $\tau (w_{1},w_{2})$ with high multiplicity, while the other observed differences will have substantially lower multiplicities. This mass represents, in particular, the difference between the offsets of the tags labeled with $w_{1}$ and $w_{2}$, respectively, defined by the peaks from the theoretical spectrum of *P* that correspond to the ladders of N-terminal ions of the same type. Thereby, we implicitly assume that fragmentation does produce ladders of ions leading to the tags labeled with $w_{1}$ and $w_{2}$, respectively.

**Figure 2:**
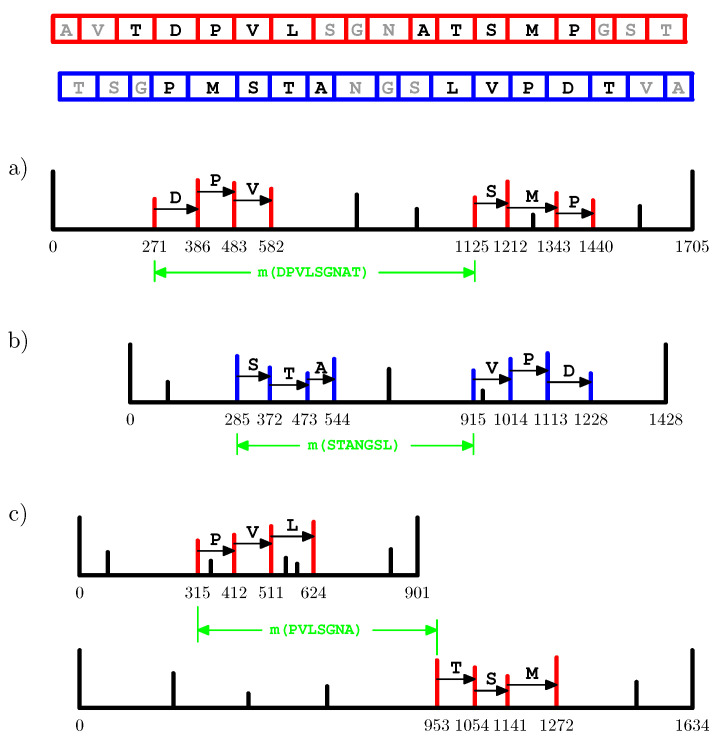
Four spectra acquired from a toy protein with the amino acid sequence AVTDPVLSGNATSMPGST. Tag convolution is being computed for the strings TDPVL and ATSMP. Two tags composing a pair that contributes the “correct” (i.e., equal to m(SGN)) value can be derived from the following: (**a**) a spectrum acquired from the entire protein; (**b**) a spectrum acquired from a fragment of the underlying protein; (**c**) two distinct spectra acquired from possibly different protein fragments starting at a same amino acid residue.

An important point is that even the spectra with very few peaks or an incorrect precursor mass, which could be neither interpreted de novo nor identified by means of a database search (should an appropriate database be available), may give rise to tags that will contribute to the “correct” offset difference (Figure 2a,b). In addition, such a pair of tags can originate from two distinct spectra, which potentially may be acquired from different—although starting at a same residue of the underlying sequence—peptides (Figure 2c). Thus, tag convolution makes a remarkably extensive use of the information encapsulated in the input dataset, capturing the details commonly missed by existing tools for analyzing MS/MS data.

However, in practice, $w_{1}$ and/or $w_{2}$ may happen not to be unique with respect to the protein sequence(s) contained in a sample, and, if so, pairs of tags corresponding to their non-correlated occurrences may produce an irrelevant offset difference endowed with a convincingly high multiplicity. A straightforward method for preventing such appearances of such fraud values comprises an appropriate selection of tag length *k*, which should then be large enough to ensure that a *k*-mer is unlikely to occur more than once in the sequence(s) being analyzed (note that an occurrence of its reversed copy would also count). Nevertheless, usage of short tags is often beneficial, despite the fact that they can be duplicated: for instance, 3-tags turn out to be particularly handy in analyzing poorly covered regions of the underlying sequence(s). On the other hand, it is often clear from the context which offset differences are more likely correct, and then incorrect values can be safely ignored regardless of their associated multiplicities. For example, if seeking to decide whether a sequence *s* represents a correct de novo interpretation of an input spectrum (see also Section 2.3 and Section 3.2), for two *k*-mers $w_{1}$ and $w_{2}$ defined as above, we would expect $Mass(a_{i}\ldots a_{j-1})$ to show up with high multiplicity. If this is the case, but some other values occur with comparably high, or even higher, multiplicities, their presence can be attributed to the fact that at least one of $w_{1}$ and $w_{2}$ occurred at least once more (possibly in a reversed form) in the sequences of the peptides contained the sample.

Another issue to be taken into account is that even for a highest-quality dataset, there is little hope to encounter a tag for *every* *k*-mer in the protein sequence(s). In order to overcome potential complications caused by the absence of some tags, we extend the concept of tag convolution from *k*-mers to longer strings, and the procedures outlined below capitalize on this generalization.

In order to define tag convolution for strings, we need to introduce two auxiliary operations that apply to tag convolution for *k*-mers. The first one is a shift by a value of δ, which transforms $\tau (w_{1},w_{2})$ into the set

$$\tau_{\delta }\left(w_{1},w_{2}\right)=\left\{\left(d+\delta ,m\right)|\left(d,m\right)\in \tau \left(w_{1},w_{2}\right)\right\}.$$

The second operation is a merge of the outputs of tag convolution for two pairs of *k*-mers; typically, at least one of those will be appropriately shifted so that the two sets of offset differences would presumably match each other. For example, merging the outputs of $\tau (w_{1},w_{2})$ and $\tau_{\delta }\left(u_{1},u_{2}\right)$ comprises merging the respective two sets of offset differences; for a difference that occurs in both sets, its multiplicity in the resulting set $\tau \left(w_{1},w_{2}\right)\circ \tau_{\delta }\left(u_{1},u_{2}\right)$ is calculated as the sum of those in the original sets, while a difference contained in precisely one set simply inherits its corresponding multiplicity.

For two amino acid strings $s_{1}=a_{1}\ldots a_{p}$ and $s_{2}=b_{1}\ldots b_{q}$, each of length at least *k*, bottom-up tag convolution $T(s_{1},s_{2})$ is computed as follows. First, for each pair of *k*-mers $w_{1}=a_{i}\ldots a_{i+k-1}$ and $w_{2}=b_{j}\ldots b_{j+k-1}$ from $s_{1}$ and $s_{2}$, respectively, where 1≤i≤p−k+1 and 1≤j≤q−k+1, we let $\delta =-Mass\left(a_{i}\ldots a_{p}\right)-Mass\left(b_{1}\ldots b_{j-1}\right)$ and compute $\tau_{\delta }\left(w_{1},w_{2}\right)$. Then, $\tau (s_{1},s_{2})$ is formed by merging all obtained sets. Subsequently, we consider the reversed copies $\bar{s_{1}}$ and $\bar{s_{2}}$ of $s_{1}$ and $s_{2}$, respectively, and compute $\tau (\bar{s_{2}},\bar{s_{1}})$ in a similar manner. Finally, we let $T\left(s_{1},s_{2}\right)=\tau \left(s_{1},s_{2}\right)\circ \tau \left(\bar{s_{2}},\bar{s_{1}}\right)$. It follows from the definition that $T\left(s_{1},s_{2}\right)=T\left(\bar{s_{2}},\bar{s_{1}}\right)$.

Assuming that $s_{1}$ and $s_{2}$ are substrings of *s* and $s_{1}$ precedes $s_{2}$ in *s*, let $s^{*}$ denote the substring of *s* separating $s_{1}$ and $s_{2}$. Then, $\tau_{\delta }\left(w_{1},w_{2}\right)$ essentially provides us with a set of weighted estimates of the mass $Mass(s^{*})$ of $s^{*}$ computed from $w_{1}$ and $w_{2}$, and $T(s_{1},s_{2})$ combines them all together, thus providing such set of estimates obtained from the entire strings $s_{1}$ and $s_{2}$ and their reversed copies $\bar{s_{1}}$ and $\bar{s_{2}}$. Suppose we trust correctness of $s_{1}$ and $s_{2}$ but doubt that of $s^{*}$. Then, the presence of $Mass(s^{*})$ in $T(s_{1},s_{2})$ with a high multiplicity would serve as an argument that $s^{*}$ is correct, while its absence from $T(s_{1},s_{2})$ or occurrence in $T(s_{1},s_{2})$ with a low multiplicity would be a “warning alarm.” This simple idea underlies the sequence validation procedures outlined in the next section.

Observe that the above-mentioned drawback of using short tags is significantly reduced for tag convolution applied to long enough amino acid strings $s_{1}$ and $s_{2}$. Indeed, even though for a pair of *k*-mers $w_{1}$ and $w_{2}$ cut out from $s_{1}$ and $s_{2}$, or $\bar{s_{2}}$ and $\bar{s_{1}}$, respectively, an *incorrect* offset difference may dominate in $\tau (w_{1},w_{2})$, it is unlikely that the same value will also appear with a high multiplicity in the output of tag convolution for other pairs of *k*-mers contributing to $T(s_{1},s_{2})$. On the contrary, the correct value should be produced with a relatively high multiplicity for *each* pair of *k*-mers from $s_{1}$ and $s_{2}$, or $\bar{s_{2}}$ and $\bar{s_{1}}$, respectively, that both belong to K; consequently, they are expected to dominate in $T(s_{1},s_{2})$.

### 2.3. Sequence Validation

It is not uncommon that the amino acid strings generated by a de novo sequencing algorithm contain erroneous amino acids or even are entirely wrong. We propose the following method for validating de novo strings using bottom-up tag convolution.

Let $s=a_{1}\ldots a_{n}$ be an amino acid string subject to validation. With each amino acid $a_{g}$ of *s* except for the first and last *k* ones, we associate its *tag score* $\theta (a_{g})$, where k<g≤n−k. With each amino acid $a_{h}$ of *s*, we associate its *k-mer score*$\kappa (a_{h})$, where 1≤h≤n.

The tag score $\theta (a_{g})$ equals the multiplicity of $Mass(a_{g})$ in the tag convolution $T(s_{l},s_{r})$ of the two substrings $s_{l}$ and $s_{r}$ of *s* located immediately to the left and right of $a_{g}$, respectively. It should be noted, however, that the farther the two *k*-mers, $w_{1}$ and $w_{2}$, are from each other within *s*, the less accurate the output of $\tau (w_{1},w_{2})$ might be, and consequently, the contribution of the pair ($w_{1}$,$w_{2}$) to $T(s_{l},s_{r})$ results. In order to prevent potential errors introduced by such pairs of tags, we impose an upper bound *L* on the length of $s_{l}$ and $s_{r}$, thus permitting $s_{l}=a_{\max\{0,g-L\}}\ldots a_{g-1}$ and $s_{r}=a_{g+1}\ldots a_{\min\{n,g+L\}}$.

The *k*-mer score of an amino acid of *s* can be either 0 or 1. Initially, all of those are set to zero. Now, suppose that at time of calculation of $\theta (a_{g})$, a pair $w_{1}=a_{i}\ldots a_{i+k-1}$ and $w_{2}=a_{j}\ldots a_{j+k-1}$ of *k*-mers from $s_{l}$ and $s_{r}$, respectively, contributed the value of $Mass(a_{g})$ to $T(w_{1},w_{2})$, where 0≤i≤g−k and g<j≤n−k+1. On one hand, this boosts confidence in $a_{g}$; on the other hand, this also favours the amino acids composing $w_{1}$ and $w_{2}$. To recognize this fact, the *k*-mer score of each of $a_{i},\ldots ,a_{i+k-1},a_{j},\ldots a_{j+k-1}$, if still zero, is risen to 1.

**Figure 3:**
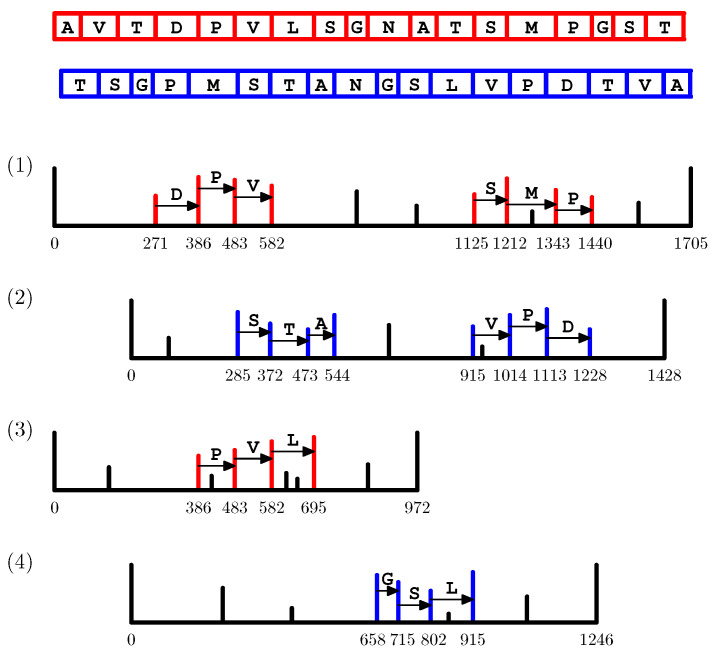
Four spectra acquired from a toy protein with the amino acid sequence *s* = AVTDPVLSGNATSMPGST together give rise to five 3-tags. The pairs of tags labeled with DPV and SMP, PVL and SMP, STA and VPD, and STA and GSL, respectively, contribute to the tag and 3-mer scores of certain amino acids of *s*.

As an example, consider a toy protein with the amino acid sequence *s* = AVTDPVLSGNATSMPGST from which four spectra were acquired (see Figure 3). The red and blue peaks correspond to *b*-ions and *y*-ions, respectively. In total, there are are six 3-tags, out of which three are based on *b*-ions (those labeled with DPV, SMP, and PVL, respectively) and the other three are based on *y*-ions (those labeled with STA, VPD, and GSL, respectively). For each amino acid of *s*, the tag and 3-mer score calculated from those 3-tags are listed in Table 1. In particular, the amino acid score of N-10 is obtained as the multiplicity of its mass 114 in the output of T(AVTDPVLSG,ATSMPGST). Since 114 occurs precisely once in the output of the following:

- $\tau_{-Mass(\mathrm{DPVLSG})-Mass(\mathrm{AT})}\left(\mathrm{DPV},\mathrm{SMP}\right)$;
- $\tau_{-Mass(\mathrm{PVLSG})-Mass(\mathrm{AT})}\left(\mathrm{PVL},\mathrm{SMP}\right)$;
- $\tau_{-Mass(\mathrm{STA})-Mass(\mathrm{GSL})}\left(\mathrm{STA},\mathrm{VPD}\right)$;
- $\tau_{-Mass(\mathrm{STA})}\left(\mathrm{STA},\mathrm{GSL}\right)$.

**Figure 4:**
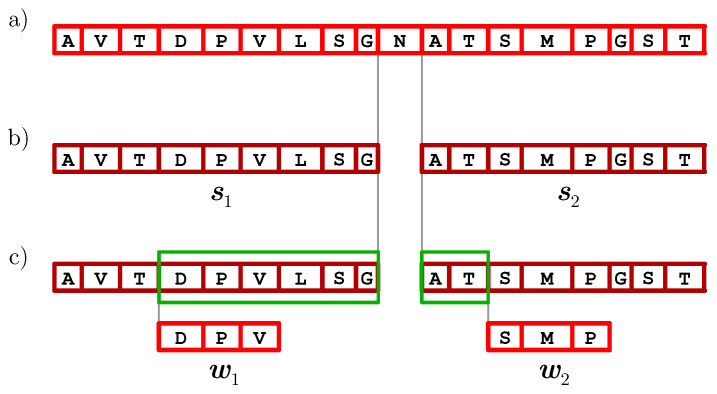
Contribution of a pair of 3-mers to T(AVTDPLSG,ATSMPGST) computed at the time of validating the amino acid N-10 of the toy protein from Figure 2. (**a**) The protein sequence. (**b**) To validate N-10, we consider the prefix $s_{1}$ and suffix $s_{2}$ of the entire sequence immediately preceding and following N-10, respectively, and examine all the pairs of 3-mers from $s_{1}$ and $s_{2}$, respectively, and from $\bar{s_{2}}$ and $\bar{s_{1}}$, respectively. (**c**) Processing of the pair of 3-mers $w_{1}$ = DPV and $w_{2}$ = SMP from $s_{1}$ and $s_{2}$, respectively. To either 3-mer, precisely one 3-tag from the set T depicted in Figure 3 corresponds. Both tags are defined by *b*-ions and properly align against the sequence. Thus, the difference between their offsets, which contributes to $\tau (w_{1},w_{2})$, equals Mass(DPVLSGNAT). When shifting this value by δ=−Mass(DPVLSG)−Mass(AT), we obtain the mass of N equal to 114. Consequently, the pair (DPV,SMP) of 3-mers contributes to T(AVTDPLSG,ATSMPGST) with a value of 114 with multiplicity 1.

The amino acid score on N-10 equals 4 (see Figure 3 and Figure 4). Furthermore, the tag score of N-10 is 0: This can be deduced immediately since it is not covered by any 3-tag. On the contrary, the tag score of each amino acid covered by some tag that together with another one contributed to the amino score of N-10 (namely, D-4, P-5, V-6, L-7, S-8, G-9, A-11, T-12, S-13, M-14, and P-15) can be immediately set to 1.

For a small enough tag length *k*, the introduced scores of the amino acids composing a correct string *s* usually are all positive, except for the *k*-mer score of the middle amino acid $a_{k+1}$ of a string *s* of length 2k+1, which is necessarily zero (while in this case, $a_{k+1}$ is the only amino acid of *s*, for which the tag score is defined). Should a few similar interpretations have been proposed, e.g., for some spectrum, incorrect interpretations occasionally may also possess this property; however, the correct one will typically have a larger sum of the tags scores of its amino acids.

## 3. Results

### 3.1. Datasets

We benchmarked our algorithms on bottom-up datasets acquired from carbonic anhydrase 2 (CAH2) and alemtuzumab [32]; brief details are provided below.

**CAH2** solution was reduced with dithiothreitol (DTT), alkylated with iodoacetamide, digested overnight with trypsin, GluC or Lys-C, and analyzed using a nanoLC system coupled to a Thermo Q-Exactive mass spectrometer. MS and MS/MS spectra were collected at a resolution of 70,000 and 17,500, respectively. In total, 177,741 HCD MS/MS spectra were acquired (trypsin: 91,747 spectra; GluC: 43,026 spectra; Lys-C: 42,968 spectra).

**Alemtuzumab** solution was reduced with DTT, alkylated with iodoacetamide, digested overnight with trypsin, proteinase K or pepsin, and analyzed by a nanoLC system coupled with a Thermo LTQ Orbitrap XL mass spectrometer. MS spectra were collected at a resolution of 15,000. For every precursor, both HCD and a CAD iontrap spectra were recorded; HCD MS/MS spectra were collected at a resolution of 7500. In total, 3695 pairs of HCD and CAD MS/MS spectra were collected (trypsin: 1358 spectra; proteinase; K: 1052 spectra; pepsin: 1285 spectra). Only HCD MS/MS spectra were used to compute tag convolution and perform de novo sequence validation.

### 3.2. Sequence Validation

The input spectra were deconvoluted with MS-Deconv [27] using the default parameters and preprocessed; the latter amounted to reflecting peaks and merging nearby ones, as described in [30]. Subsequently, we applied the Twister approach [30], initially developed for the top-down case, to generate from them a set of *de novo* strings, and through searching those with BLAST against the non-redundant database, again following [30], detected and identified 32 and 2 contaminants in the CAH2 and alemtuzumab sample, respectively. The lists of contaminants are provided in Appendix A and Appendix B.

Subsequently, we ran PepNovo+ [33,34,35] on either dataset, with the fragment and precursor mass tolerance of 0.01 and 0.05 Da, respectively, and a fixed post-translational modification C+57. For CAH2 and alemtuzumab, 55,156 and 2471 spectra were thereby interpreted, respectively, in up to 20 ways each. A total of 806,934 and 38,936 de novo sequences of length at least seven were generated for CAH2 and alemtuzumab, respectively, among which 90,891 and 1765 were correct, respectively (i.e., represented a sequence fragment of either a target protein or contaminant).

Furthermore, we generated from either dataset a set of 3-tags as described in Section 2.1 using the mass tolerance of ε=4 mDa. The obtained 419,136 and 7945 3-tags for CAH2 and alemtuzumab, respectively, were then used by the sequence validation procedure to evaluate de novo strings. When comparing the values output by tag convolution with the corresponding amino acid masses, we used an error tolerance of 0.02 Da.

When validating the de novo strings, we first restricted our attention to those with associated scores that are all positive. Next, for each spectrum, we sorted such strings (if any) by decreasing sum of the tag scores of their amino acids and iteratively eliminated for each string *s* all the subsequent strings $s^{\prime }$ such that the following is the case:

- $Length\left(s^{\prime }\right)\leq Length\left(s\right)+1$;
- The best alignment of $s^{\prime }$ against *s* resulted in the Hamming distance of at most 2 between the matched fragments for all the alignments satisfying the following conditions:
  (a) If $Length\left(s^{\prime }\right)\leq Length\left(s\right)$, $s^{\prime }$ must be matched against a substring of *s* with length $Length(s^{\prime })$;
  (b) Otherwise (i.e., if $Length\left(s^{\prime }\right)=Length\left(s\right)+1$), *s* must be matched against either the prefix or suffix of $s^{\prime }$ with length Length(s);
  (c) Thereby, neither insertions nor deletions were allowed.

Here, Length(s) and $Length(s^{\prime })$ denotes the length of the string *s* and $s^{\prime }$, respectively. As a final step, all the strings of length 7 with the middle tag score less than *h* were eliminated. For CAH2, the threshold *h* was set to 300, implying that approximately 37.67% of the sequences having length 7 were retained. However, for alemtuzumab, since the number of 3-tags was pretty small, we set *h* to 1 so that all the strings of length 7 still under consideration actually were retained.

In this manner, we were left with 104,211 and 1559 sequences for CAH2 and alemtuzumab, respectively, among which 79,451 and 1323 were correct, respectively. Thus, approximately 87.41% and 74.96% of the correct sequences were retained for CAH2 and alemtuzumab, respectively, while the fraction of those (in a corresponding set) increased from 11.26% and 4.53% to 76.24% and 84.86%, respectively.

The detailed statistics on the de novo strings generated from either dataset are provided in Table 2.

### 3.3. The TagConvolution Software Tool

The proposed approach was implemented in a Java tool TagConvolution, which is freely available at http://bioinf.spbau.ru/en/twister/tag-convolution accessed on 8 November 2021, along with the sample input and output files.

The program takes as input two directories: one storing the file(s) containing the deconvoluted with MS-Deconv tandem mass spectra, which will be used by the validation procedure for tag generation, and the other—the file(s) with the amino acid sequences to be validated. The sequence files are either generated as output by PepNovo+ [34] or contain lists of candidate interpretations of the input spectra in a very simple format illustrated in the sample file TagConvolutionSampleInput.txt.

The tag generation strategy is the same as those used within the Twister de novo sequencing approachs [30,36]. Consequently, the TagConvolution tool inherits the following input parameters of Twister: the tag length *k*, the mass tolerance applied when retrieving tags, and two flags indicating whether peak reflection and water-loss peak elimination should be performed. Further details can be found in [30].

Moreover, the mass tolerance used by the sequence validation procedure when matching tag convolution values to the respective amino acid masses and the threshold on the minimum tag score of the middle amino acid in a string of length (2k+1) are specified.

For each input file InputFileName.txt, two output files InputFileName.valid.txt and InputFileName.scores.txt are produced. For each MS/MS spectrum, at least one interpretation of which was classified as valid, all such candidate sequences are listed in the former file, and their associated tag and *k*-mer scores are provided in the latter.

The TagConvolution tool performs quite fast: in particular, on a modern laptop, the entire CAH2 dataset was processed in approximately 90 s.

## 4. Discussion

We have introduced the concept of tag convolution and demonstrated its utility in validating candidate tryptic peptide sequences based on a set of bottom-up MS/MS spectra collected at a high resolution. In practice, enzymes of any specificity can be used for digesting the target protein. Neither the protein size nor the peptide amino acid composition matters after digestion. The developed method can process sets of CID/CAD, ETD/ECD, or HCD MS/MS spectra acquired from the peptides subject to analysis.

In particular, this approach represents an elegant method for verifying de novo sequencing results using the same data, from which they were derived, yet it differs in processing. The proposed procedure can be easily adapted for localizing and identifying post-translational modifications (PTMs) in proteins or peptides: If for two disjoint sequence fragments, the value with the highest multiplicity output by tag convolution is not consistent with the sum of masses of the amino acid residues in-between, this likely points to one or a few PTMs that occurred on (some of) those, and the difference between the theoretically expected and observed value can be used to characterize the putative PTMs.

Additionally, bottom-up tag convolution can be applied for appropriately gluing together overlapping aggregated strings—protein sequence fragments derived from top-down spectra as described in [30,36]—assuming that bottom-up data were collected as well. We will benefit from that to further extend the Twister algorithm for de novo sequencing of proteins.

We implemented the sequence validation procedure in a standalone computer program freely available at http://bioinf.spbau.ru/en/twister/tag-convolution accessed on 8 November 2021, along with the sample input and output for the computational experiments described in this paper (however, the underlying tag generation strategy is the same as used within Twister [30,36]). Another direction for future work can be development of a more sophisticated software system for validating and possibly correcting amino acid sequences subject to examination.

Finally, we note that top-down deconvolution tools, including MS-Deconv, may not recognize some “good” isotopic envelopes in bottom-up MS/MS spectra because they differ in shape from those in top-down spectra. Consequently, several tags present in the original spectra may become lost at time of deconvolution. Therefore, it would be beneficial to adapt the scoring function employed by MS-Deconv for evaluating candidate isotopic envelopes in the case of high-resolution bottom-up mass spectrometry data so as to further enhance reliability of the proposed approach.

## Figures and Tables

**Table 1:** The tag and 3-mer score for each amino acid of the protein sequence from the toy example provided in Figure 3.

	A	V	T	D	P	V	L	S	G	N	A	T	S	M	P	G	S	T
tag score	-	-	-	0	0	0	2	3	3	4	2	2	0	0	0	-	-	-
3-mer score	0	0	0	1	1	1	1	1	1	0	1	1	1	1	1	0	0	0

**Table 2:** Statistics on the de novo strings for CAH2 and alemtuzumab. During validation, first the strings with associated scores all that were positive (necessarily of length above 7) were selected and made subject to filtration based on the alignment procedure described in the main text. Furthermore, the strings of length 7 were handled separately, and those with the middle tag score at least *h* were selected. The strings with length above and precisely 7 were retained upon alignment-based and middle tag score-based filtration, respectively, and they composed the set of strings that passed the validation procedure. The threshold *h* on the middle tag score was set to 300 and 1 for CAH2 and alemtuzumab, respectively. The details on the strings selected at some stage of the validation procedure are highlighted in bold. The percentage of the correct strings is given with respect to the total number of strings available upon completion of the respective stage.

		CAH2	Alemtuzumab
de novo strings of length ≥7	total	806,934	38,936
	correct	90,891 (11.26%)	1765 (4.53%)
with the associated scores all positive (necessarily of length >7)	total	69,205	685
	correct	46,738 (67.54%)	592 (86.42%)
with the associated scores all zeros	total	523,382	36,569
	correct	3258 (0.62%)	285 (0.78%)
**upon filtration**			
retained	total	58,084	656
	correct	46,382 (79.85%)	582 (88.72%)
eliminated	total	11,121	29
	correct	356 (3.20%)	10 (34.48%)
**de novo strings of length 7**			
with the middle tag score ≥h	total	46,127	903
	correct	33,069 (71.69%)	741 (82.06%)
with the middle tag score <h	total	76,330	0
	correct	5673 (7.43%)	0
**final results**			
de novo strings of length ≥7 that passed the validation procedure	total	104,211	1559
	correct	79,451 (76.24%)	1323 (84.86%)

## Data Availability

The sample data sets are freely available at http://bioinf.spbau.ru/en/twister/tag-convolution accessed on 8 November 2021.

## Abbreviations

The following abbreviations are used in this manuscript:

MS/MS	Tandem mass spectrometry;
CAH2	Carbonic anhydrase 2;
DTT	Dithiothreitol;
HCD	Higher-energy C-trap dissociation;
CAD	Collisionally activated dissociation.

## Appendix A. Contaminants in the CAH2 Data Set

1. sp|P62992|1-76 Ubiquitin-40S ribosomal protein S27a [Bos taurus]
2. sp|P13696|PEBP1_BOVIN Phosphatidylethanolamine-binding protein 1 OS = Bos taurus GN = PEBP1 PE = 1 SV = 2
3. gi|27806297|ref|NP_776676.1| flavin reductase (NADPH) [Bos taurus]
4. gi|157830773|pdb|1CYO|A Chain A, Bovine Cytochrome B(5)
5. gi|6006423|emb|CAB56828.1| hemoglobin alpha chain [Bos taurus]
6. gi|77735367|ref|NP_001029380.1| ribonuclease UK114 [Bos taurus]
7. gi|27807109|ref|NP_777040.1| superoxide dismutase [Cu-Zn] [Bos taurus]
8. gi|296480569|tpg|DAA22684.1| TPA: thymosin, beta 4-like [Bos taurus]
9. gi|149642641|ref|NP_001092620.1| D-dopachrome decarboxylase [Bos taurus]
10. gi|28189771|dbj|BAC56500.1| similar to peptidylprolyl isomerase A (cyclophilin A), partial [Bos taurus]
11. gi|29135329|ref|NP_803482.1| glutathione S-transferase P [Bos taurus]
12. gi|114051361|ref|NP_001039513.1| selenium-binding protein 1 [Bos taurus]
13. gi|59858077|gb|AAX08873.1| aspartate aminotransferase 1 [Bos taurus]
14. gi|61888856|ref|NP_001013607.1| triosephosphate isomerase [Bos taurus]
15. gi|27806591|ref|NP_776501.1| glutathione peroxidase 1 [Bos taurus]
16. gi|75057676|sp|Q58DC0.1|CPPED_BOVIN RecName: Full = Serine/threonine-protein phosphatase CPPED1; AltName: Full = Calcineurin-like phosphoesterase domain-containing protein 1
17. gi|134085635|ref|NP_001076965.1| lactoylglutathione lyase [Bos taurus]
18. gi|62751849|ref|NP_001015572.1| protein DJ-1 [Bos taurus]
19. gi|27819608|ref|NP_776342.1| hemoglobin subunit beta [Bos taurus]
20. gi|114051487|ref|NP_001039526.1| cytochrome c [Bos taurus]
21. gi|229552|prf||754920A albumin [Bos taurus]
22. gi|77736203|ref|NP_001029800.1| malate dehydrogenase, cytoplasmic [Bos taurus]
23. gi|58760467|gb|AAW82141.1| NDP kinase NBR-A [Bos taurus]
24. gi|77735583|ref|NP_001029487.1| adenosylhomocysteinase [Bos taurus]
25. gi|94966811|ref|NP_001035592.1| alpha-1-acid glycoprotein precursor [Bos taurus]
26. gi|78365305|ref|NP_001030533.1| peptidyl-prolyl cis-trans isomerase FKBP1A [Bos taurus]
27. gi|27807167|ref|NP_777068.1| peroxiredoxin-6 [Bos taurus]
28. gi|48428343|sp|Q7M135.1|LYSC_LYSEN RecName: Full = Lysyl endopeptidase; AltName: Full=Lys-C
29. gi|136429|sp|P00761.1|TRYP_PIG RecName: Full=Trypsin; Flags: Precursor
30. gi|914833|gb|AAB60696.1| keratin type II, partial [Homo sapiens]
31. gi|386854|gb|AAA36153.1| type II keratin subunit protein, partial [Homo sapiens]
32. gi|623409|gb|AAA60544.1| keratin 10 [Homo sapiens]

## Appendix B. Contaminants in the Alemtuzumab Data Set

1. gi|224977|prf||1205229A proteinase K
2. gi|136429|sp|P00761.1|TRYP_PIG RecName: Full = Trypsin; Flags: Precursor
